# Higher relevance of mechanical determinants for short-distance performance and metabolic determinants for middle-distance performance in female adolescent swimmers at national level

**DOI:** 10.1038/s41598-025-92056-y

**Published:** 2025-02-28

**Authors:** Sebastian Keller, Patrick Wahl

**Affiliations:** 1 Section Exercise Physiology, German Sport University Cologne, Cologne, Germany; 2 The German Research Centre of Elite Sport, German Sport University Cologne, Cologne, Germany; 3 German Sport University Cologne, Am Sportpark Müngersdorf 6, 50933 Cologne, Germany

**Keywords:** Performance determinants, Strength training, Performance diagnostics, Lactate threshold, Maximal oxygen uptake, Correlation, Physiology, Scientific data

## Abstract

The study investigated associations of metabolic, anthropometric, and neuromuscular parameters with 50 to 400 m front crawl performance. Competition performances of 24 female swimmers (14.9 ± 1.3 years) were recorded and metabolic determinants (maximal oxygen uptake and lactate accumulation [ċLa_max_], cost of swimming [C], and lactate threshold 1 [LT1] using 200 m all-out, 20 s sprint, 500 m submaximal, and 3 min incremental test, respectively), anthropometry and dryland strength (squat and bench press 1 repetition maximum [1RM_SQ_/1RM_BP_] and mean propulsive power [MPP_SQ_/MPP_BP_]) were assessed. 1RM_SQ_ (61.9 ± 13.3 kg) and MPP_BP_ (207 ± 45 W) correlated significantly with 50 (1.84 ± 0.07 m∙s^−1^) and 100 m performance (1.68 ± 0.06 m∙s^−1^) (*r* ≥ 0.45) and ċLamax (0.35 ± 0.12 mmol·L^−1^·s^−1^) and body mass (60.1 ± 7.0 kg) with 50 and 100 m, respectively (*r* ≥ 0.44). Only LT1 (1.23 ± 0.04 m∙s^−1^) correlated significantly with 200 (1.52 ± 0.05 m∙s^−1^) and 400 m performance (1.43 ± 0.06 m∙s^−1^) (*r* ≥ 0.56). Multiple regression explained 33–35% and 61–86% of the variance in short- and middle-distance performance based on 1RM_SQ_ and arm span and LT1, C, and fat percentage, respectively. Based on the analyses, mechanical determinants are more predictive of short- and metabolic determinants of middle-distance performance.

## Introduction

Swimming performance, i.e., the highest average swimming speed achieved in competition, is determined by a complex interplay of metabolic power, including anaerobic and aerobic energy contributions, and energy cost of swimming (C), which is influenced by mechanical parameters, e.g., anthropometric, neuromuscular, and technical properties^1,2^. Therefore, diagnostic assessments frequently involve parameters to estimate the development and/or contributions of those determinants to performance^3^^–^^5^. These include for example maximal lactate accumulation assessed during short all-out tests for anaerobic power^4,6^, maximal oxygen uptake (V̇O_2peak_) during incremental tests or time trials for aerobic power^3,7,8^, and oxygen consumption (V̇O_2_) during swimming at a given speed as a measure of C^9,10^ on the metabolic side. In addition, metabolic (e.g., lactate) thresholds are also frequently assessed as they present the interplay of metabolic power input and C^5,9^. On the mechanical side, anthropometric characteristics (including body dimensions and composition) and dryland strength (i.e., maximal strength and power) are commonly evaluated^11–13^.

To find out which of these determinants might be most important, various studies have related diagnostic parameters to swimming performance. In front crawl swimming, for example, the correlation between V̇O_2peak _and performance has been frequently investigated for short distances, i.e., 50 and 100 m (e.g.,^4,7,14^), and middle distances, i.e., 200 and 400 m (e.g.,^8,9,15,16^). Interestingly, the individual studies not only reported differences between distances, which would have been expected, e.g., due to different energy system contributions^7^ but also within the same distance. Thus, correlation coefficients ranged from *r* = 0.02 to *r*= 0.54 for short-distance performance^4^^,7^^,17^ and from *r* = 0.30 to *r*= 0.70 for middle-distance performance^8^^,9^^,15^^,16,^ which raises the question of where this variance originates from. Potential explanations include different testing times across the season, varying test protocols, and measurement or parameter determination methods.

Besides such methodological discrepancies, heterogeneous sample characteristics in terms of sex, age, and performance level are a further source of random variance that limits the comparability of the study findings. In addition, heterogeneity within a studied sample can also have a strong impact on the resulting correlation coefficients^18^. This could be the case, for example, when pooling data from male and female swimmers where sex-specific differences, e.g., lower dryland strength and V̇O_2peak _as well as performance, are expected^7,11,14^ and may lead to overestimation of the correlation coefficient.

Unfortunately, studies investigating associations between metabolic and mechanical determinants and performance in male and female swimmers separately or even only in female swimmers are scarce and focus, if at all, on early junior swimmers (i.e., age between 10 and 14 years)^13,19,20^. However, since the late junior age (i.e., 15–17 years) is much more predictive of high performance in adulthood^21,22^, it would be even more important to know which determinants are related to performance in this age group. In one of the few studies that examined female late junior swimmers at a high level, Unnithan et al.^10^ found no statistically significant correlations between V̇O_2peak _and performance from 50 to 1000 m, which contradicts some studies conducted in highly trained men showing statistically significant moderate to high correlations, at least with middle-distance performance (e.g.,^16,23^). Regarding anthropometric and neuromuscular parameters, Seffrin et al.^12^ reported no statistically significant correlation for body height, mass, composition, and segment lengths, as well as handgrip strength and squat or countermovement jump performance with 100 to 400 m performance in female late junior swimmers, but this could be at least partially related to the small sample size of *N* = 4, which seems underpowered. Besides the cited studies, to the best of the authors’ knowledge, there is currently no study that comprehensively investigates the associations of several metabolic and mechanical parameters with performance over different race distances among female late junior athletes at a high level.

Given this paucity of evidence, in this study we systematically examined the relationships of various commonly assessed metabolic (i.e., blood lactate accumulation, V̇O_2peak_, C, and LT1), anthropometric (i.e., body height, mass, composition, and arm span), and neuromuscular parameters (i.e., squat and bench press maximal strength and power), with front crawl swimming performance over different race distances in a homogeneous group of female late junior swimmers at the national level. By always using the same test protocols and assessment methods and using race times over multiple distances collected during the competition period from the same participants, we aimed to overcome some of the methodological issues associated with the equivocal literature findings shown previously. Thus, we sought to identify which metabolic and mechanical parameters are most important for short- and middle-distance front crawl performance in this specific group.

## Results

Of the 24 athletes included, 21, 24, 22, and 19 swimmers obtained official race results for 50, 100, 200, and 400 m freestyle, respectively (Table 1), yielding mean speeds of 1.84 ± 0.07, 1.68 ± 0.06, 1.52 ± 0.05, and 1.43 ± 0.06 m·s^−1^, respectively. In addition, Table 1 presents all descriptive values for performance as well as the metabolic and mechanical determinants including slight variations in sample size due to missing race times, invalid spirometric measurements (2 x for V̇O_2peak_ and 1 x for C), determination of LT1 (2 x), and bioimpedance measurements (2 x for body fat percentage), and problems in performing the neuromuscular tests with adequate exercise technique (3 x for bench press and 4 x for squat).

**Table 1:** Descriptive performance data as well as metabolic and mechanical characteristics of participants presented as mean ± standard deviation along with the range and coefficient of variation (CV).

	*n*	Mean ± SD	Range	CV [%]
**Performance time**				
50 m [s]	21	27.2 ± 1.1	25.8–29.5	4.0
100 m [s]	24	59.4 ± 2.1	55.9–64.6	3.6
200 m [s]	22	131.4 ± 4.4	121.9–137.4	3.3
400 m [s]	19	279.4 ± 11.7	262.5–299.0	4.2
**Metabolic**				
V̇O_2peak_ [L·min^−1^]	22	3.48 ± 0.37	2.91–4.28	10.7
La_peak_ [mmol·L^−1^]	24	6.70 ± 1.66	4.19–9.99	24.7
ċLa_max_ [mmol·L^−1^·s^−1^]	24	0.35 ± 0.12	0.16–0.58	33.3
C [mL·m^−1^]	23	35.4 ± 5.9	22.4–48.6	16.6
LT1 [m·s^−1^]	22	1.23 ± 0.04	1.16–1.29	2.9
**Anthropometric**				
Height [cm]	24	172 ± 7	161–184	4.0
Body mass [kg]	24	60.1 ± 7.0	48.4–73.0	11.7
Fat percentage [%]	22	17.1 ± 4.3	9.0–26.4	25.0
Arm span [cm]	20	176 ± 9	164–200	5.0
**Neuromuscular**				
1RM_BP_ [kg]	21	48.2 ± 7.8	35.5–65.0	16.3
MPP_BP_ [W]	21	207 ± 45	122–286	21.8
1RM_SQ_ [kg]	20	61.9 ± 13.3	37.0–87.5	21.5
MPP_SQ_ [W]	20	265 ± 64	161–366	24.3

Abbreviations: n: sample size; V̇O_2peak_: maximal oxygen uptake; La_peak_: peak blood lactate concentration; ċLa_max_: maximal lactate accumulation rate; C: energy cost of swimming; LT1: lactate threshold 1; 1RM_BP_: bench press one repetition maximum; MPP_BP_: bench press maximal mean propulsive power; 1RM_SQ_: squat one repetition maximum; MPP_SQ_: squat maximal mean propulsive power.

**Fig. 1:**
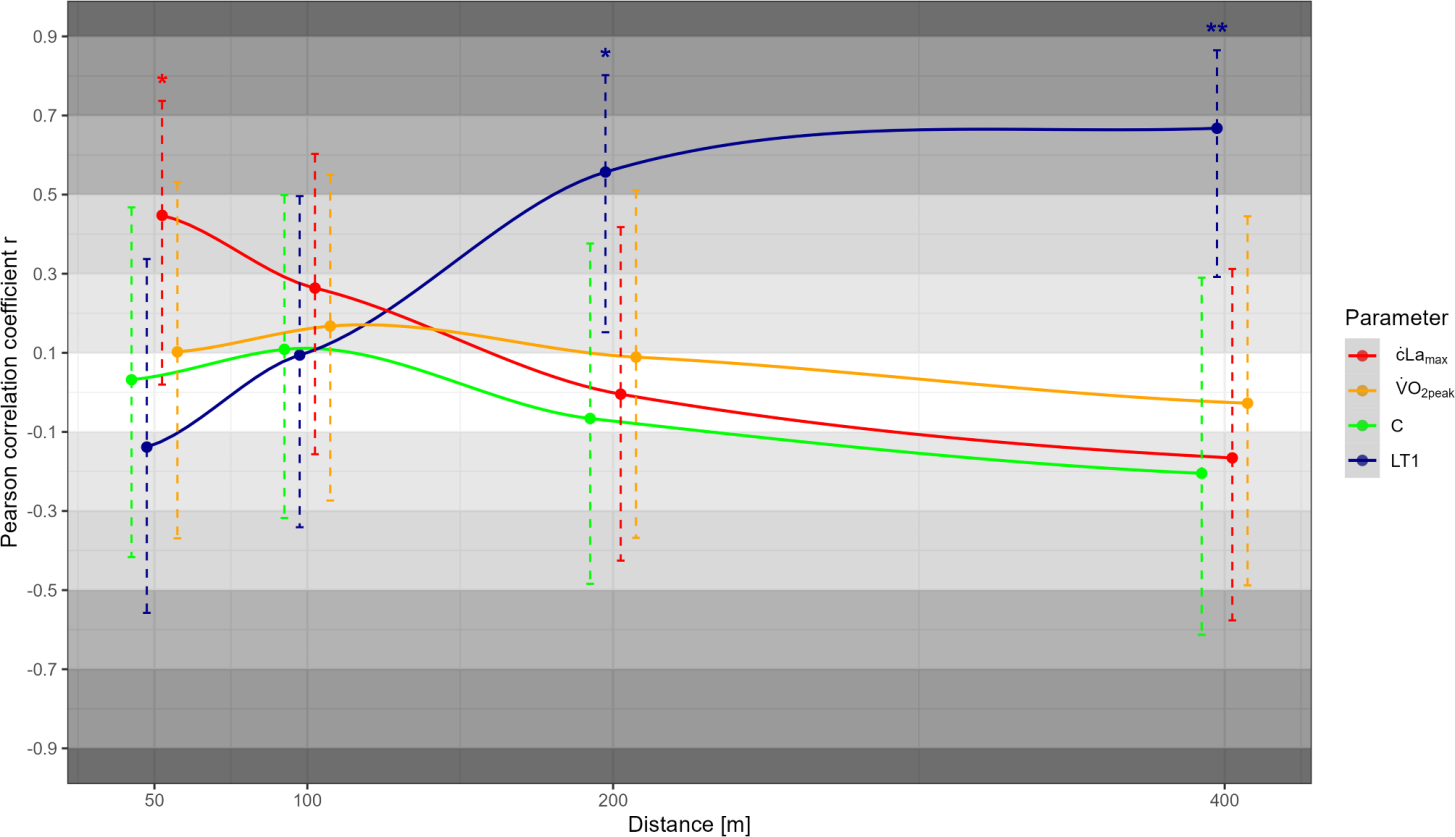
Pearson correlation coefficients *r* along with the 95% confidence interval for the metabolic parameters, maximal lactate accumulation rate (ċLa_max_), maximal oxygen uptake (V̇O_2peak_), energy cost of swimming (C), and lactate threshold 1 (LT1) with performance (i.e., average speed) across the race distances. Statistically significant correlations are indicated by ^*^ *p* < 0.05 and ^**^ *p* < 0.01.

**Fig. 2:**
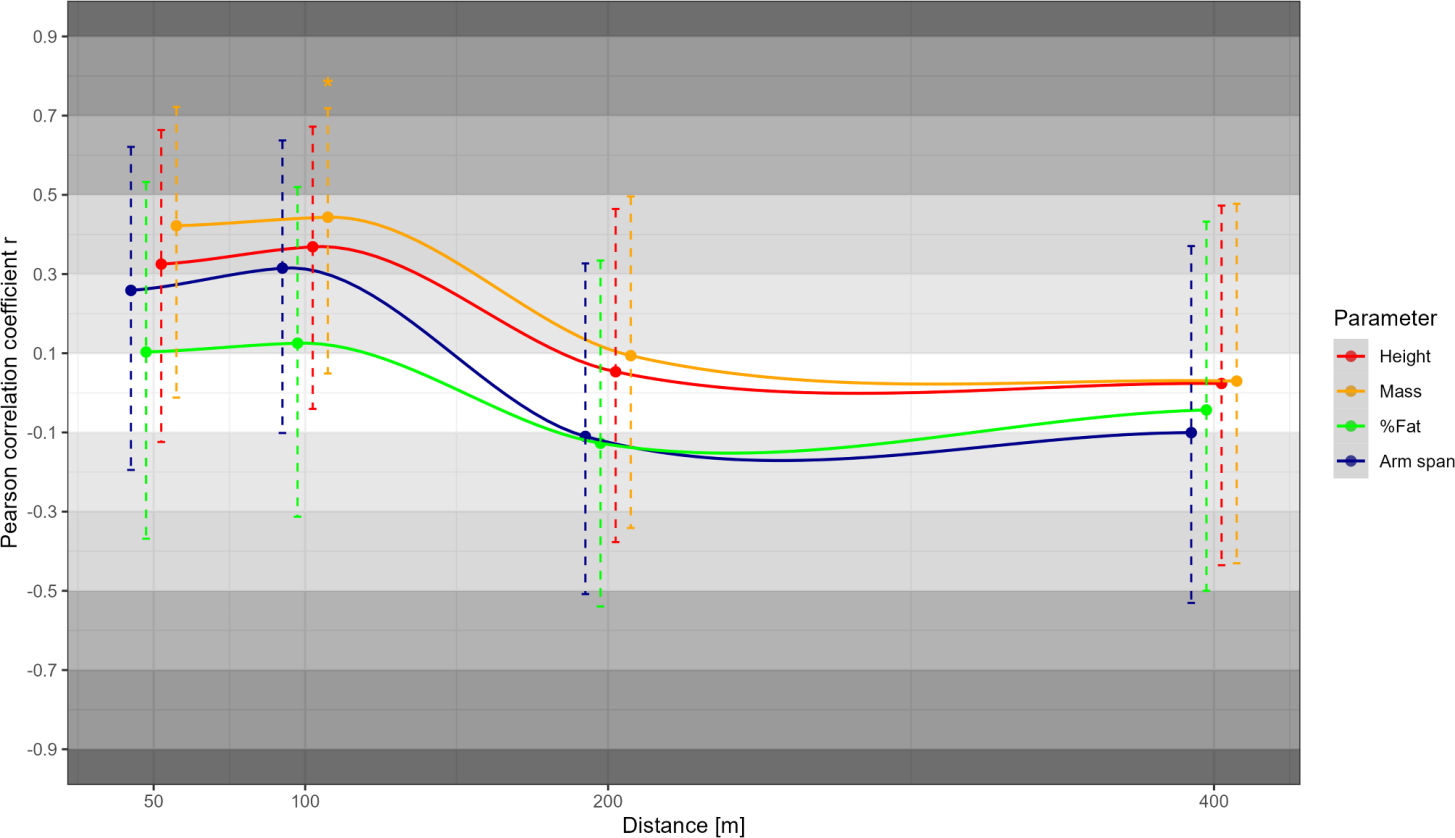
Pearson correlation coefficients *r* along with the 95% confidence interval for the anthropometric parameters, body height, mass, fat percentage (%Fat), and arm span with performance (i.e., average speed) across the race distances. Statistically significant correlations are indicated by ^*^ *p* < 0.05.

**Fig. 3:**
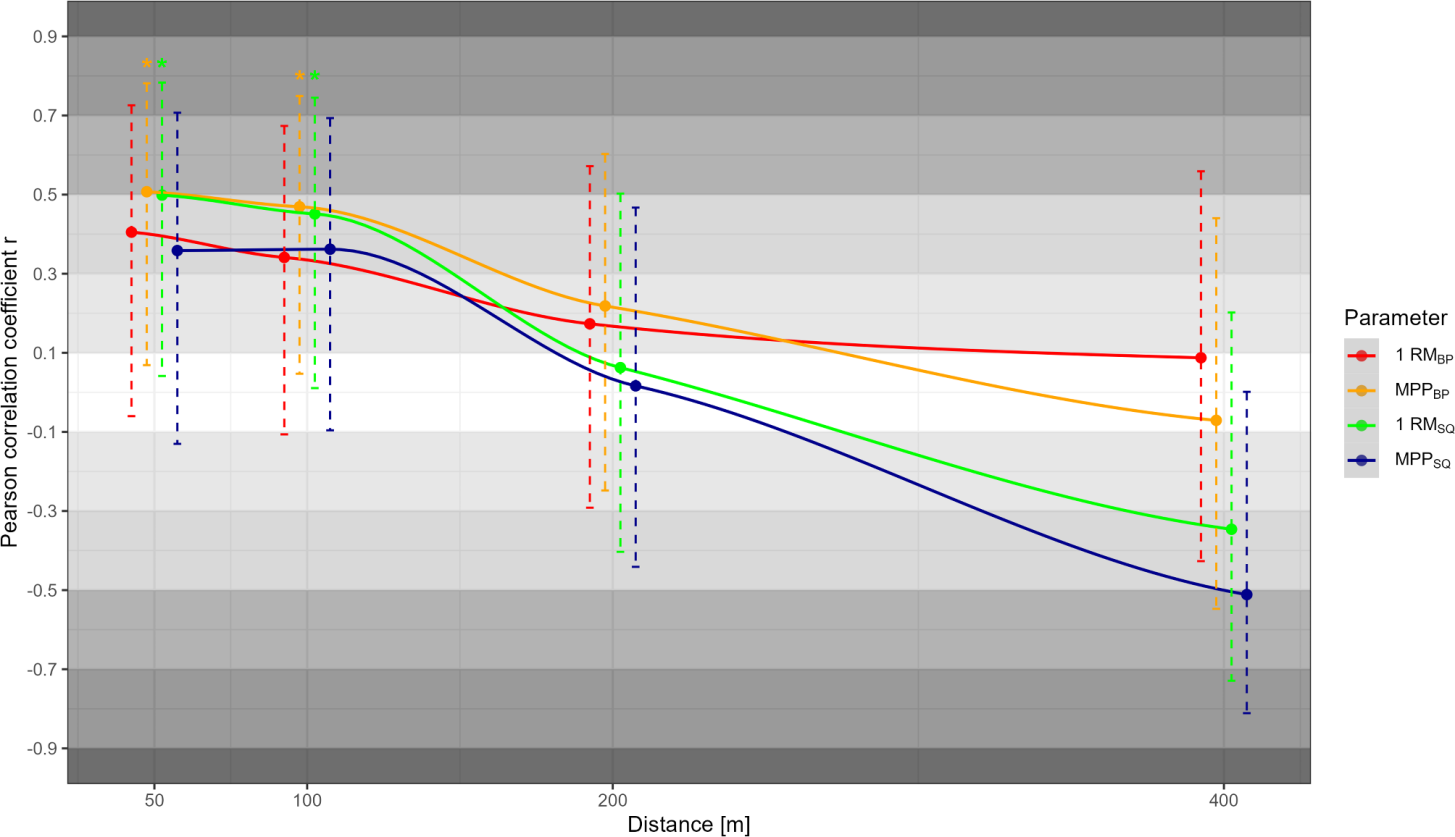
Pearson correlation coefficients *r* along with the 95% confidence interval for the neuromuscular parameters, bench press 1 repetition maximum (1RM_BP_), bench press mean propulsive power (MPP_BP_), squat 1 repetition maximum (1RM_SQ_), squat mean propulsive power (MPP_SQ_) with performance (i.e., average speed) across the race distances. Statistically significant correlations are indicated by ^*^ *p* < 0.05.

**Fig. 4:**
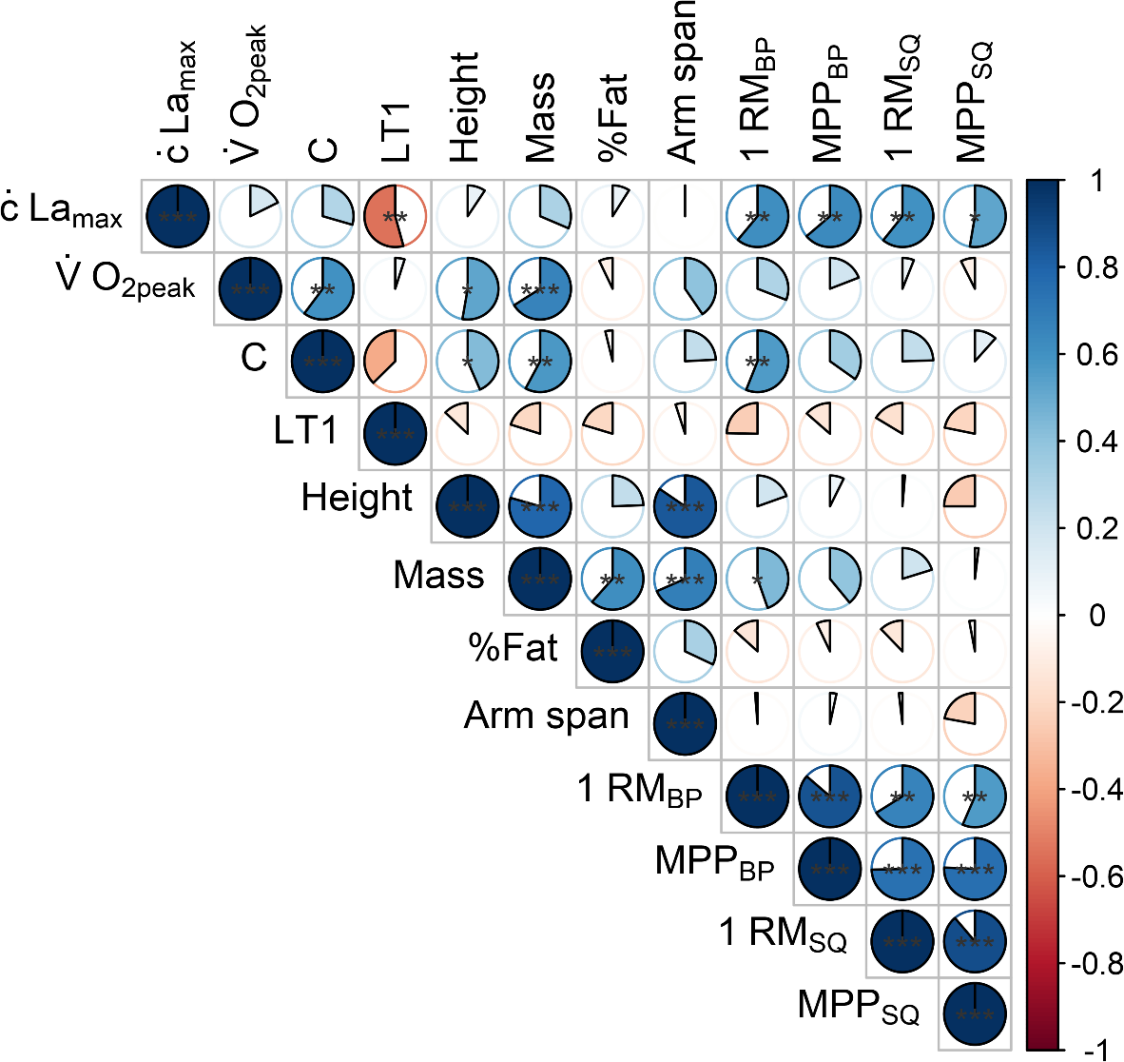
Pearson correlation coefficients *r* between metabolic (maximal lactate accumulation rate [ċLa_max_], maximal oxygen uptake [V̇O_2peak_], energy cost of swimming [C], and lactate threshold 1 [LT1]), anthropometric (body mass, height, fat percentage [%Fat] and arm span), and neuromuscular (bench press 1 repetition maximum [1RM_BP_], bench press mean propulsive power [MPP_BP_], squat 1 repetition maximum [1RM_SQ_], and squat mean propulsive power [MPP_SQ_]) performance determinants. Statistically significant correlations are indicated by ^*^ *p* < 0.05, ^**^ *p* < 0.01, and ^***^ *p* < 0.001.

Correlations of metabolic, anthropometric, and neuromuscular determinants with swimming performance over the different race distances are presented in Figs. 1, 2, and 3 respectively. Of the metabolic parameters, ċLa_max_ correlated moderately with 50 m performance (*r* = 0.45, *p* = 0.04), while LT1 correlated highly with middle-distance performance (*r* ≥ 0.56, *p* ≤ 0.01). Due to consistently lower correlations between La_peak_ and performance compared to ċLa_max_, only the latter was shown in Fig. 2. In contrast to moderate to high correlations between most anthropometric variables (i.e., height, mass, and arm span; *r* ≥ 0.26) and short-distance performance, trivial to low correlations were observed for the middle distances (−0.13 ≤ *r* ≤ 0.09). All neuromuscular parameters were moderately to highly associated with short-distance performance (*r* ≥ 0.34), while trivial to small positive or even moderate to high negative correlations were observed for 200 and 400 m performance (*r* ≤ 0.22). Besides correlations with performance, Fig. 4 shows the correlations between all metabolic and mechanical performance determinants.

The findings of the multiple regression analysis are presented in Table 2. Similar to the bivariate correlations, mechanical parameters (i.e., 1RM_SQ_ and arm span) were retained in the models for short-distance performance, whereas metabolic parameters (i.e., LT1 and C) were primarily retained for middle-distance performance.

**Table 2:** Model summary resulting from stepwise multiple regression analyses using the performance (average speed) across the different race distances as dependent variable and the metabolic (maximal lactate accumulation rate [ċLa_max_], maximal oxygen uptake [V̇O_2peak_], energy cost of swimming [C], and lactate threshold 1 [LT1]), anthropometric (body mass, height, fat percentage [%Fat] and arm span), and neuromuscular (bench press 1 repetition maximum [1RM_BP_], bench press mean propulsive power [MPP_BP_], squat 1 repetition maximum [1RM_SQ_], and squat mean propulsive power [MPP_SQ_]) performance determinants as independent variables.

Distance (*n*)	Predictor	Beta	SE	Std. beta	t	*p*	*R* ^2^	Adj. *R*^2^	F	*p*
**50 m** (*n* = 16)	(Intercept)	1.657	0.079	–	21.057	< 0.001	0.30	0.25	6.098	0.03
	1RM_SQ_	0.003	0.001	0.551	2.469	0.03				
**100 m** (*n* = 18)	(Intercept)	1.197	0.235	–	5.100	< 0.001	0.35	0.27	4.104	0.04
	1RM_SQ_	0.002	0.001	0.489	2.354	0.03				
	Arm span	0.002	0.001	0.315	1.514	0.15				
**200 m** (*n* = 17)	(Intercept)	1.055	0.274	–	3.847	0.002	0.61	0.51	6.656	0.006
	LT1	0.492	0.194	0.489	2.537	0.03				
	%Fat	−0.004	0.002	−0.384	−2.077	0.06				
	C	−0.002	0.001	−0.297	−1.557	0.14				
**400 m** (*n* = 14)	(Intercept)	0.761	0.243	–	3.126	0.01	0.85	0.80	18.23	< 0.001
	LT1	0.708	0.178	0.529	3.991	0.003				
	%Fat	−0.000	0.000	−0.449	−3.443	0.006				
	C	−0.004	0.002	−0.317	−2.465	0.03				

Abbreviations: n: sample size; Beta: beta coefficient; SE: standard error; Std. beta: standardized beta coefficient; R^2^: coefficient of determination, Adj. R^2^: adjusted coefficient of determination.

## Discussion

The aim of the present study was to investigate which metabolic and mechanical parameters are most important for short- and middle-distance front crawl performance in a homogeneous group of female adolescent swimmers at the national level. Overall, mechanical parameters were more important for short-distance performance, while metabolic parameters contributed most to middle-distance performance. Specifically, 1RM_SQ_ and MPP_BP_ were significantly correlated with 50 and 100 m performance (*r* ≥ 0.45) and ċLa_max_ and body mass with 50 and 100 m, respectively (*r* ≥ 0.44), while LT1 was the only parameter significantly related to 200 and 400 m performance (*r *≥ 0.56). By combining the predictive power of the single parameters using multiple regression, 33–35% and 61–86% of the variance in short- and middle-distance performance could be explained. Despite various studies reporting associations between metabolic or mechanical determinants and swimming performance (e.g.,^3,4,11–14^), we aimed to overcome at least some of the methodological issues associated with previous inconclusive results by always using the same test protocols and assessment methods and by using race times over multiple distances collected from the same participants during the competition period. Due to the high and homogenous performance level of our participants, we are not surprised that overall correlations are lower than in the previous studies with lower-level or more heterogeneous samples (see above).

For example, in agreement with the findings from Unnithan et al.^10^, but in strong contradiction to other studies (e.g.,^3,14^), we found low non-significant correlations between V̇O_2peak_ and swimming performance across all distances (i.e., –0.03 ≤ r ≤ 0.17), which is likely due to the homogeneous high level. Thus, it was suggested early on in swimming, but also in other sports, that at a high performance level, when approaching a certain physiological limit for V̇O_2peak_, submaximal indicators such as LT1 become more predictive of performance^9,24^. Therefore, even though V̇O_2peak_ and C represent the main determinants of swimming performance from a theoretical point of view^1^, they showed no relevant correlations with middle-distance performance. In contrast, LT1 was strongly associated with middle-distance performance, which is in line with previous work^5,25^. Taken together, this indicates that different combinations of V̇O_2peak_ and C (e.g., relatively high V̇O_2peak_ together with high C or relatively low V̇O_2peak_ and C) can result in high LT1 and thus middle-distance performance^5,9^. This is further underlined by the high positive and statistically significant correlation between V̇O_2peak_ and C, which is also in line with previous work^5^. Regarding the short distances, we found a moderate and statistically significant positive correlation between ċLa_max_ and 50 m performance, consistent with previous work in high-level adolescent to adult swimmers of both sexes^6^, reflecting the importance of anaerobic glycolytic energy supply for short exercise times (i.e., 25.8–29.5 s)^17^. The lower correlation coefficient in the present compared to the previous study could be based on a lower variation in 50 m performance, i.e., 4.0% vs. 11.6%, which is likely due to the inclusion of different strokes and both sexes^6^.

Among the mechanical determinants, anthropometrics generally showed the lowest associations with performance (*r *≤ 0.44). Nonetheless, the consistently low to moderate correlations of body height, mass, and arm span emphasize a certain role of body dimensions for short-distance swimming. While larger height and arm span may be related to a more favorable streamline position or leverage ratio for generating high propulsive forces^26,27^, body mass in this cohort can be considered similar to lean mass and therefore also directly related to propulsive force production^27^, which is crucial for sprint performance^2,28^. This is also reflected in the moderate to large correlations between MPP_BP_ and 50 and 100 m performance, consistent with previous work (e.g.,^5,11,29^), as bench press strength can be considered a good dryland estimate for front crawl thrust^30^. In contrast, moderate, statistically significant positive correlations between 1RM_SQ_ and short-distance performance are likely mediated by the considerable influence of the start (e.g., 12–27% for short-course sprint performance^31^), which has been shown highly associated with lower-body dryland strength^29,32^. Unexpectedly, however, despite the increasing relevance of turns with increasing race distance^31^, we found moderate negative correlations between lower-body strength and middle-distance performance. A possible explanation could be that swimmers with stronger legs also adopt a more leg-pronounced swimming style, which is assumed to require more energy and therefore be less efficient, especially with increasing race distance^33,34^.

Interestingly, besides moderate to large positive correlations with sprint performance, all dryland strength parameters also demonstrated highly positive and statistically significant associations with ċLa_max_, indicating a common underlying mechanism, such as skeletal muscle mass and/or fiber type distribution. Thus, both higher muscle mass or type II proportion could be related to higher glycolytic power and dryland strength^27,35^. This explanation is further supported by the highly negative and statistically significant association between ċLa_max_ and LT1 since as a submaximal indicator of aerobic performance, the latter is rather related to the oxidative (i.e., fiber type I dominant) phenotype^24^.

In addition to the bivariate correlations, multiple regression, which allowed testing which parameter combinations might be most predictive for performance, demonstrated that mechanical determinants (i.e., 1RM_SQ_ and arm span) were more important for short-distance performance, whereas middle-distance performance was mainly dependent on metabolic determinants (i.e., LT1, C, and body fat percentage). However, while the majority of the variance (i.e., 61–85%) in middle-distance performance was explained by the model, only 30–35% could be explained in short-distance swimming. This could be related, among other things, to the non-specific dryland tests used, which differ from the force application in water despite high correlations with thrust (e.g., due to the lack of hydrostatic pressure and drag on dryland)^5,36^. Alternatively, the rather conservative statistical approach (i.e., using AIC as the criterion for the stepwise procedure instead of F-tests) may have led to a lower variance explanation since the goodness of the model fit (i.e., variance explanation) is balanced against model complexity (i.e., number of model predictors). Despite the limited predictive value of the multiple regression analyses (especially for short-distance performance), the results can still be used to draw training derivations for this specific group, which has been largely underrepresented in the literature so far. Thus, while national-level female adolescent short-distance swimmers should focus more on strength development, middle-distance swimmers benefit from a well-developed aerobic basis.

The following aspects should be considered when interpreting the study results. Due to the strict inclusion criteria (i.e., female adolescents with a high and homogeneous performance level for the front crawl with current race times over 50 to 400 m), which is considered a strength of the present compared to previous studies, the sample size is limited and also varies slightly across the different race distances, limiting the multiple regression analysis. The limited sample size is also the reason why more sophisticated statistical approaches, including machine learning based algorithms (e.g.,^37^) could not be used in the present study and may therefore be an interesting area for future research. In addition, we did not control for menstrual cycle phase, which could theoretically have an impact on our results. However, since current study findings estimate the influence to be minor^38^, we deliberately decided against controlling for the menstrual cycle phase, as this is also not taken into account in competitions so far and therefore our approach reflects the real-world settings. Lastly, due to the demanding testing program in combination with relatively short resting periods (i.e., ≥ 15 min), it cannot be excluded that recovery may have been compromised, potentially affecting the diagnostic results. However, as all participating athletes had been used to competing at national level for at least two years, they appeared to be familiar with such schedules^39^, so this influence is considered minor.

In conclusion, moderately positive and statistically significant correlations were observed for 1RM_SQ_, MPP_BP_, ċLa_max_, and body mass with 50 and 100 m performance, while LT1 was the only parameter that was highly positively and significantly associated with 200 and 400 m performance in national-level female adolescent swimmers. Based on these correlations combined with the results of the multiple regression analyses, short-distance swimmers should focus more on neuromuscular training and middle-distance swimmers on aerobic swimming training.

## Methods

### Participants

Twenty-four healthy, female adolescent swimmers (age: 14.9 ± 1.3 yrs, range: 12.9–18.1 yrs, coefficient of variation [CV]: 9.0%) volunteered to participate in the study. They were all members of the highest squad in their age group and had at least 2 years of competition experience at the national level. Their average seasonal water and dryland training volumes amounted to 13.1 ± 2.9 and 3.2 ± 1.0 h·wk^−1^, respectively, and all athletes had been systematically performing heavy barbell training for ≥ 1 year. Based on their seasonal best performance, 14 of them were categorized as short-distance (50/100 m) and 10 as middle-distance (200/400 m) swimmers specialized mainly in front crawl (17), but few also in backstroke (4), and individual medley (2), and butterfly (1). Nevertheless, all athletes regularly competed in freestyle competitions at the national level. According to their seasonal best performance, which amounted to 721 ± 43 World Aquatic Points (range: 659–797, CV: 6.0%) and following the classification proposed by Ruiz-Navarro et al.^40^, they were all assigned to performance level 3. All athletes and their parents were informed about the benefits and risks of the investigation and provided written informed consent. The study was conducted according to the declaration of Helsinki and was approved by the local ethical committee (125/2020).

### Study design

In this study, swimmers were tested as part of a longitudinal observational study at the peak of the competition period. Therefore, the study design and test protocols are presented in detail elsewhere^5^. Briefly, athletes were instructed to refrain from intensive training 24 h preceding the diagnostic assessment and to standardize nutritional intake using a 24 h diet record. On the testing day, an anthropometric assessment and a 20 s sprint test were performed first, followed by a submaximal 500 m test, 200 m all-out test, and a submaximal 3-min incremental step test, each in water, as well as two dryland incremental strength tests, which were all completed in randomized order. A rest period of ≥ 15 min and ≥ 30 min was ensured between all water tests and between water and dryland tests, respectively. All water tests were completed on the lateral lane of a 50 m pool using the front crawl technique, in-water starts, and flip turns. During the submaximal swimming tests, swimming speeds were prescribed using a visual pacing device (Virtual Swim Trainer, Indico Technologies, Torino, Italy; precision: 0.02 s). Heart rate was measured using chest straps (HRM-Swim™, Garmin Deutschland GmbH, Garching, Germany) and 20 µl of capillary blood for lactate analysis (Biosen C-line; EKF Diagnostic Sales, Magdeburg, Germany) was collected from the dried earlobe at specific time points indicated below.

### Procedures

Dryland tests included the assessment of anthropometrics, i.e., body height, mass, and composition (seca 274, seca GmbH & Co KG, Hamburg, Germany), as well as arm span, which was always performed by the same experienced diagnostician using an anthropometric tape. In addition, incremental strength tests were performed to determine athletes’ load-velocity profiles in squat and bench press exercises using a Smith machine (Gym80 international GmbH, Gelsenkirchen, Germany). After a standardized warm-up, the testing protocol started with the unloaded bar (22 kg) as the initial load and was individually increased as described previously^5^. For each load, the trial with the highest mean propulsive power (MPP, determined during the concentric part of the movement using a linear velocity transducer with a sampling rate of 1000 Hz [T-Force System, Ergotech, Murcia, Spain]) was used to compute the load-velocity profiles. The highest MPP value measured across all loads was termed MPP_SQ_ and MPP_BP_ and the repetition with the highest load performed safely and without assistance was considered the one repetition maximum (1RM_SQ_ and 1RM_BP_) for squat and bench press, respectively.

After an individualized warm-up on land and in water (~ 15 min), athletes first performed the 20 s sprint test using in-water starts from a prone position with foot contact to the pool wall on an acoustic signal until they were stopped by an acoustic and a tactile signal. Capillary blood samples were taken immediately before and until the 9^th^ minute after the sprint (every minute) to determine peak blood lactate concentration (La_peak_) and maximal lactate accumulation rate (ċLa_max_) as estimates of anaerobic power^5,6^. ċLa_max_ was calculated according to Eq. (1) with La_pre_ as blood lactate concentration determined immediately before the test, t_exerc_ as total exercise time (20 s) and t_alac_ as theoretical alactic time, which was set to 4 s according to Heck et al.^41^.

1 $$\:{\dot{c}La}_{max}=\frac{{La}_{max}-\:{La}_{pre}}{{t}_{exer}-\:{t}_{alac}}$$

C and V̇O_2peak_ were assessed via gas exchange measurement after a 500 m submaximal swimming bout at 1.2 m·s^−1^ (this speed was chosen for comparability with previous studies assessing C in young female swimmers^10^^,33^) and 200 m all-out, respectively (MetaMax 3B, Cortex Biophysik GmbH, Leipzig, Germany). The 500 m submaximal swim was divided into 300 and 200 m, with a 1 min rest in between to verify steady state conditions by repeated V̇O_2_ measurement, and the 200 m all-out was preceded by 100 m at progressively increased speed to ensure sufficient exercise time for V̇O_2peak_ attainment. For the determination of both C and V̇O_2peak_, end-exercise V̇O_2_ was estimated based on post-exercise V̇O_2_ measurement corrected for the decline in heart rate, as this technique has been shown to allow the highest agreement with online V̇O_2_ measurement^42,43^. The highest 5 s average of the corrected V̇O_2_ was considered for analysis, respectively^5^. C was determined as the average of the repeated V̇O_2 _measurement (i.e., after 300 and 200 m submaximal swimming) divided by speed^5^, also taking into account the metabolic energy derived from lactate production (by multiplying the net value of lactate accumulation after both laps by the oxygen equivalent for lactate accumulation in blood of 2.7 mL·mmol^−1^·kg^−1^)^44^.

Further, a submaximal incremental step test was conducted to determine LT1, starting at 88% of the current 400 m best performance minus 4 steps (0.12 m·s^−1^) and increasing by 0.03 m·s^−1^ every 3 min (plus 40 s resting periods for blood sampling in between)^5,25^. In contrast to previous work, the incremental test was not performed until exhaustion but was terminated when an increase in blood lactate levels of > 0.4 mmol·L^−1^ was observed in combination with a rating of perceived exertion ≥ 15, which was sufficient to determine LT1. In accordance with previous work^5,25^, LT1 was defined as the point on the blood lactate speed curve fitted with a third-order polynomial with a slope equal to 10 (i.e., corresponding to a theoretical increase in blood lactate concentration of 0.3 mmol·L^−1^ per step). Due to the almost perfect correlation between swimming speed at LT1 (determined with this method) and maximal lactate steady state as previously demonstrated (i.e., *r *= 0.97)^25^, LT1 was considered an adequate estimate of aerobic capacity, allowing the incremental test to be kept submaximal to limit the overall effort for the athletes.

Around the time of the diagnostic assessment (average time difference: ± 25 days), competition results of all participants in official long-course freestyle races from 50 to 400 m were collected using the publicly accessible database (Swimrankings.net, https://www.swimrankings.net/, Splash Software Ltd., Spiegel bei Bern, Swiss) of the European Swimming Association (Ligue Européenne de Natation). The race times were then converted into average speeds in [m·s^−1^], which were used as dependent variables for further analyses (see below).

### Statistical analysis

Statistical analysis was performed using the *stats* package in R (Version 4.3.1, R Core Team, 2023), with an alpha level of 0.05 applied for all statistical tests. All descriptive values are expressed as mean ± standard deviation (SD), range, and CV, the latter calculated as a measure of homogeneity by dividing the SD by the mean. After visual inspection of normal distribution and homoscedasticity using Q-Q and residual plots, Pearson product-moment correlation coefficients *r* along with the 95% confidence interval were used to examine the relationships of metabolic (La_peak_, ċLa_max_, V̇O_2peak_, C, and LT1), anthropometric (i.e., body height, mass, composition, and arm span), and neuromuscular parameters (1RM_SQ_, 1RM_BP_, MPP_SQ_, and MPP_BP_) with competition performance over the different distances (50, 100, 200, and 400 m). Correlation coefficients were classified as trivial (*r* < 0.1), low (0.1 ≤ *r* < 0.3), moderate (0.3 ≤ *r* < 0.5), high (0.5 ≤ *r* < 0.7), very high (0.7 ≤ *r* < 0.9), and nearly perfect (*r *≥ 0.9) according to Hopkins^45^.

Further, to investigate the combined influence of the metabolic and mechanical determinants (independent variables) on performance (dependent variable), multiple regression analysis using a bi-directional stepwise selection procedure based on the Akaike information criterion (AIC) was conducted separately for each race distance. The final models were checked for multicollinearity using the variance inflation factor (*car* package) with a cut-off set to 5.

## Data Availability

The datasets generated and analyzed during the current study are available from the corresponding author on reasonable request.

## References

[1] Zamparo, P., Cortesi, M. & Gatta, G. The energy cost of swimming and its determinants. *Eur. J. Appl. Physiol.* **120**, 41–66 (2020). 31807901 10.1007/s00421-019-04270-y

[2] Ruiz-Navarro, J. J. et al. Factors relating to sprint swimming performance: A systematic review. *Sport Med.*10.1007/s40279-024-02172-4 (2025). 10.1007/s40279-024-02172-4 PMC12011652 39841367

[3] Jürimäe, J. et al. Analysis of swimming performance from physical, physiological, and Biomechanical parameters in young swimmers. *Pediatr. Exerc. Sci.* **19**, 70–81 (2007). 17554159 10.1123/pes.19.1.70

[4] Lätt, E. et al. Physiological, Biomechanical and anthropometrical predictors of sprint swimming performance in adolescent swimmers. *J. Sport Sci. Med.* **9**, 398–404 (2010). PMC3761703 24149633

[5] Keller, S. et al. Development and interplay of metabolic and mechanical performance determinants over an annual training period in adolescent national-level squad swimmers. *Int. J. Sports Physiol. Perform.* **18**, 1398–1411 (2023). 37730208 10.1123/ijspp.2023-0072

[6] Mavroudi, M., Kabasakalis, A., Petridou, A. & Mougios, V. Blood lactate and maximal lactate accumulation rate at three sprint swimming distances in highly trained and elite swimmers. *Sports* **11**, 87 (2023). 37104161 10.3390/sports11040087 PMC10146159

[7] Papoti, M. et al. Aerobic and anaerobic performances in tethered swimming. *Int. J. Sports Med.* **34**, 712–719 (2013). 23382009 10.1055/s-0031-1291250

[8] Lätt, E. et al. Physical development and swimming performance during biological maturation in young female swimmers. *Coll. Antropol*. **33**, 117–122 (2009). 19408614

[9] Ribeiro, J. P. et al. Metabolic predictors of middle-distance swimming performance. *Br. J. Sports Med.* **24**, 196–200 (1990). 2078807 10.1136/bjsm.24.3.196 PMC1478791

[10] Unnithan, V. et al. Aerobic cost in elite female adolescent swimmers. *Int. J. Sports Med.* **30**, 194–199 (2009). 19199194 10.1055/s-0028-1104583

[11] Chalkiadakis, I., Arsoniadis, G. G. & Toubekis, A. G. Dry-land force–velocity, power–velocity, and swimming-specific force relation to single and repeated sprint swimming performance. *J. Funct. Morphol. Kinesiol.* **8**, 120 (2023). 37606415 10.3390/jfmk8030120 PMC10443377

[12] Seffrin, A., Delira, C. A., Nikolaidis, P. T., Knechtle, B. & Andrade, M. S. Age-related performance determinants of young swimmers in 100- and 400-m events. *J. Sports Med. Phys. Fit.* **62**, 9–18 (2022). 10.23736/S0022-4707.21.12045-6 33586935

[13] Sokołowski, K. et al. Biological age in relation to somatic, physiological, and swimming kinematic indices as predictors of 100 m front crawl performance in young female swimmers. *Int. J. Environ. Res. Public. Health*. **18**, 6062 (2021). 34199894 10.3390/ijerph18116062 PMC8200104

[14] Nagle, E. F. et al. Reliability and validity of a pool-based maximal oxygen uptake test to examine High-intensity short-duration freestyle swimming performance. *J. Strength. Cond Res.* **33**, 1208–1215 (2019). 31034459 10.1519/JSC.0000000000003113

[15] Costa, M. J., Santos, C. C., Marinho, D. A., Silva, A. J. & Barbosa, T. M. Modelling the 200 m front-crawl performance predictors at the winter season peak. *Int. J. Environ. Res. Public. Health*. **17**, 2126 (2020). 32210037 10.3390/ijerph17062126 PMC7142514

[16] Reis, J. F., Alves, F. B., Bruno, P. M., Vleck, V. & Millet, G. P. Oxygen uptake kinetics and middle distance swimming performance. *J. Sci. Med. Sport*. **15**, 58–63 (2012). 21802360 10.1016/j.jsams.2011.05.012

[17] Almeida, T. A. F. et al. V˙ O 2 kinetics and energy contribution in simulated maximal performance during short and middle distance-trials in swimming. *Eur. J. Appl. Physiol.* **120**, 1097–1109 (2020). 32212025 10.1007/s00421-020-04348-y

[18] Hassler, U. & Thadewald, T. Nonsensical and biased correlation due to pooling heterogeneous samples. *J. R Stat. Soc. Ser. D Stat.* **52**, 367–379 (2003).

[19] Geladas, N. D., Nassis, G. P. & Pavlicevic, S. Somatic and physical traits affecting sprint swimming performance in young swimmers. *Int. J. Sports Med.* **26**, 139–144 (2005). 15726490 10.1055/s-2004-817862

[20] Saavedra, J. M., Escalante, Y. & Rodríguez, F. A. A multivariate analysis of performance in young swimmers. *Pediatr. Exerc. Sci.* **22**, 135–151 (2010). 20332546 10.1123/pes.22.1.135

[21] Post, A. K., Koning, R. H., Visscher, C. & Elferink-Gemser, M. T. Tracking performance and its underlying characteristics in talented swimmers: a longitudinal study during the junior-to-senior transition. *Front. Physiol.* **14**, 1221567 (2023). 37621763 10.3389/fphys.2023.1221567 PMC10446966

[22] Post, A. K., Koning, R. H., Visscher, C. & Elferink-Gemser, M. T. Multigenerational performance development of male and female top-elite swimmers–A global study of the 100 m freestyle event. *Scand. J. Med. Sci. Sport*. **30**, 564–571 (2020). 10.1111/sms.13599 PMC7028091 31725946

[23] Sousa, A. C. et al. V̇O2 kinetics in 200-m race-pace front crawl swimming. *Int. J. Sports Med.* **32**, 765–770 (2011). 21913155 10.1055/s-0031-1279772

[24] Coyle, E. F. Improved muscular efficiency displayed as tour de France champion matures. *J. Appl. Physiol.* **98**, 2191–2196 (2005). 15774697 10.1152/japplphysiol.00216.2005

[25] Keller, S., Manunzio, C. & Wahl, P. Comparison of different test protocols to determine maximal lactate steady state intensity in swimming. *J. Sci. Med. Sport*. **25**, 696–701 (2022). 35667961 10.1016/j.jsams.2022.05.012

[26] Alves, M., Carvalho, D. D., Fernandes, R. J. & Vilas-Boas, J. P. How anthropometrics of young and adolescent swimmers influence stroking parameters and performance? A systematic review. *Int. J. Environ. Res. Public. Health*. **19**, 2543 (2022). 35270236 10.3390/ijerph19052543 PMC8909379

[27] Nevill, A. M., Oxford, S. W. & Duncan, M. J. Optimal body size and limb length ratios associated with 100-m personal-best swim speeds. *Med. Sci. Sports Exerc.* **47**, 1714–1718 (2015). 25412299 10.1249/MSS.0000000000000586

[28] Morais, J. E., Barbosa, T. M., Gomeñuka, N. A. & Marinho, D. A. Effects of anthropometrics, thrust, and drag on stroke kinematics and 100 m performance of young swimmers using path-analysis modeling. *Scand. J. Med. Sci. Sport*. **34**, e14578 (2024). 10.1111/sms.14578 38389142

[29] Keiner, M. et al. The influence of upper- and lower-body maximum strength on swim block start, turn, and overall swim performance in sprint swimming. *J. Strength. Cond Res.* **35**, 2839–2845 (2021). 31425457 10.1519/JSC.0000000000003229

[30] Morais, J. E., Marques, M. C., Rodríguez-Rosell, D., Barbosa, T. M. & Marinho, D. A. Relationship between thrust, anthropometrics, and dry-land strength in a National junior swimming team. *Phys. Sportsmed.* **48**, 304–311 (2020). 31787067 10.1080/00913847.2019.1693240

[31] Born, D. P., Kuger, J., Polach, M. & Romann, M. Turn fast and win: the importance of acyclic phases in top-elite female swimmers. *Sports* **9**, 122 (2021). 34564327 10.3390/sports9090122 PMC8472918

[32] West, D. J., Owen, N. J., Cunningham, D. J., Cook, C. J. & Kilduff, L. P. Strength and power predictors of swimming starts in international sprint swimmers. *J. Strength. Cond Res.* **25**, 950–955 (2011). 20664366 10.1519/JSC.0b013e3181c8656f

[33] Chatard, J. C., Lavoie, J. M. & Lacourl, J. R. Analysis of determinants of swimming economy in front crawl. *Eur. J. Appl. Physiol. Occup. Physiol.* **61**, 88–92 (1990). 2289503 10.1007/BF00236699

[34] Morris, K. S., Osborne, M. A., Shephard, M. E., Jenkins, D. G. & Skinner, T. L. Velocity, oxygen uptake, and metabolic cost of pull, kick, and whole-body swimming. *Int. J. Sports Physiol. Perform.* **12**, 1046–1051 (2017). 27967275 10.1123/ijspp.2016-0322

[35] Bellinger, P. et al. The muscle typology of elite and world-class swimmers. *Int. J. Sports Physiol. Perform.* **17**, 1179–1186 (2022). 35661058 10.1123/ijspp.2022-0048

[36] Muniz-Pardos, B. et al. Swim-specific resistance training: A systematic review. *J. Strength. Cond Res.* **33**, 2875–2881 (2019). 31343554 10.1519/JSC.0000000000003256

[37] Liu, C. et al. Improved prediction of swimming talent through random forest analysis of anthropometric and physiological phenotypes. *Phenomics* **4**, 465–472 (2024). 39723229 10.1007/s43657-024-00176-8 PMC11666874

[38] Taylor, M. Y. et al. Menstrual cycle phase has no influence on performance-determining variables in endurance-trained athletes: the FENDURA project. *Med. Sci. Sport Exerc.* **56**, 1595–1605 (2024). 10.1249/MSS.0000000000003447 38600646

[39] Greenwood, J. D., Moses, G. E., Bernardino, F. M., Gaesser, G. A. & Weltman, A. Intensity of exercise recovery, blood lactate disappearance, and subsequent swimming performance. *J. Sports Sci.* **26**, 29–34 (2008). 17852681 10.1080/02640410701287263

[40] Ruiz-Navarro, J. J., López-Belmonte, Ó., Gay, A., Cuenca-Fernández, F. & Arellano, R. A new model of performance classification to standardize the research results in swimming. *Eur. J. Sport Sci.* **23**, 478–488 (2023). 35193458 10.1080/17461391.2022.2046174

[41] Heck, H., Schulz, H. & Bartmus, U. Diagnostics of anaerobic power and capacity. *Eur. J. Sport Sci.* **3**, 1–23 (2003).

[42] Rodríguez, F. A., Chaverri, D., Iglesias, X., Schuller, T. & Hoffmann, U. Validity of postexercise measurements to estimate peak VO2 in 200-m and 400-m maximal swims. *Int. J. Sports Med.* **38**, 426–438 (2017). 28482368 10.1055/s-0042-123707

[43] Chaverri, D., Schuller, T., Iglesias, X., Hoffmann, U. & Rodríguez, F. A. A new model for estimating peak oxygen uptake based on postexercise measurements in swimming. *Int. J. Sports Physiol. Perform.* **11**, 419–424 (2016). 26356150 10.1123/ijspp.2015-0227

[44] Figueiredo, P., Zamparo, P., Sousa, A., Vilas-Boas, J. P. & Fernandes, R. J. An energy balance of the 200 m front crawl race. *Eur. J. Appl. Physiol.* **111**, 767–777 (2011). 20978781 10.1007/s00421-010-1696-z

[45] Hopkins, W. G. A scale of magnitudes for effect statistics. *Sportscience* **5**, 1–7 (2002). https://www.sportsci.org/resource/stats/effectmag.html

